# Austin District Medical Society

**Published:** 1892-12

**Authors:** T. J. Bennett, G. W. Christian

**Affiliations:** Secretary; President


### AUSTIN DISTRICT MEDICAL SOCIETY.

The Twenty-first Quarterly Meeting of the Austin District Medical Sociery will be held in Medical Hall, Austin, Texas, Thursday, December 22, 1892. You are invited to be present and participate in the following programme:

1. "Fractures of the Skull and their Treatment," by Dr. S. E. Hudson; discussion opened by Drs. Thos. D. Wooten and F. S. White.
2. "Cerebral Syphilis," by Dr. Matthew M. Smith; discussion opened by Drs. W. T. Richmond and C. O. Weller.
3. "Erysipelas of Infancy," by Dr. C. A. Danforth; discussion opened by Drs. J. A. Holloway and J. S. Watson.
4. Gonorrhœa," by Dr. W. J. Mathews; discussion opened by Drs. F. A. Martin and W. A. Ellison.
5. Retiring address of the President.
6. Election of Officers.
7. Voluntary Papers and Report of Cases.
8. Banquet at Billeisen's Cafè.

T. J. BENNETT, Secretary.

G. W. CHRISTIAN, President.

